# An Engineered Synthetic Pathway for Discovering Nonnatural Nonribosomal Peptides in *Escherichia coli*

**DOI:** 10.1128/mBio.01474-17

**Published:** 2017-10-10

**Authors:** Sara Cleto, Timothy K. Lu

**Affiliations:** a Department of Electrical Engineering and Computer Science, Massachusetts Institute of Technology, Cambridge, Massachusetts, USA; b Department of Biological Engineering, Massachusetts Institute of Technology, Cambridge, Massachusetts, USA; c Synthetic Biology Center, Massachusetts Institute of Technology, Cambridge, Massachusetts, USA; University of British Columbia

**Keywords:** nonribosomal peptides, pathway engineering, genome engineering, heterologous gene expression, mutasynthesis, polyamines, siderophores

## Abstract

Peptides that are synthesized independently of the ribosome in plants, fungi, and bacteria can have clinically relevant anticancer, antihemochromatosis, and antiviral activities, among many other. Despite their natural origin, discovering new natural products is challenging, and there is a need to expand the chemical diversity that is accessible. In this work, we created a novel, compressed synthetic pathway for the heterologous expression and diversification of nonribosomal peptides (NRPs) based on homologs of siderophore pathways from *Escherichia coli* and *Vibrio cholerae*. To enhance the likelihood of successful molecule production, we established a selective pressure via the iron-chelating properties of siderophores. By supplementing cells containing our synthetic pathway with different precursors that are incorporated into the pathway independently of NRP enzymes, we generated over 20 predesigned, novel, and structurally diverse NRPs. This engineering approach, where phylogenetically related genes from different organisms are integrated and supplemented with novel precursors, should enable heterologous expression and molecular diversification of NRPs.

## IMPORTANCE

Nonribosomal peptides (NRPs) constitute a source of bioactive molecules with potential therapeutic applications. However, discovering novel NRPs by rational engineering of biosynthetic pathways remains challenging. Here, we show that a synthetic compressed pathway in which we replaced biosynthetic genes with their ancestral homologs and orthologs enabled successful heterologous NRP expression. Polyamines added exogenously were incorporated into nascent NRPs, and molecular production was pressured by growing the host under conditions that make such NRPs beneficial for survival. This multilayered approach resulted in the assembly of over 20 distinct and novel molecules. We envision this strategy being used to enable the production of NRPs from heterologous pathways.

## INTRODUCTION

Natural products harvested from plants, fungi, and bacteria have extended human life expectancy and improved quality of life by treating difficult diseases such as cancer and bacterial infections (1). Many of these natural products are assembled by very large enzymes. The biosynthetic enzymes are composed of units (i.e., modules), each of which can be further divided into catalytic domains (2–4). Depending on whether these enzymes catalyze reactions where an amino acid or an α-carboxyacyl coenzyme A (CoA) is activated and condensed into nascent molecules, they generate nonribosomal peptides (NRPs) or polyketides (PKs), respectively. Less commonly, mixed-gene operons or hybrid genes can generate hybrid molecules that have both NRP and PK characteristics. Despite our historic ability to discover natural products with useful therapeutic properties, this process has become much less productive over time. This is in part due to the repeated discovery of molecules that are easily accessible and have already been characterized (5).

Researchers have attempted a multitude of approaches to access new natural products, such as discovering novel producer strains in less-exploited niches, like the human microbiome (6); activating silent gene clusters; and engineering genes, modules, domains, and pathways in heterologous, genetically tractable hosts (7). The first report on the successful assembly of new natural products by combining heterologous and unrelated biosynthetic genes dates back to 1985 (8). In this work, Hopwood et al. cloned genes coding for an assortment of PK antibiotics into several *Streptomyces* strains and successfully produced new, hybrid molecules. Others have used screening for antibiotic resistance as a means to enrich bacterial libraries for producers of specific groups of antibiotics (9). In addition, by cloning plant genes for substrate synthesis and expressing polyketide synthases and posttranslational modification enzymes, Katsuyama et al. produced plant flavonoids and stilbenes in *Escherichia coli* when providing the cell with carboxylic acids (10). Nguyen et al. modified daptomycin’s biosynthetic pathway to prevent glycosylation and altered the amino acids to be incorporated into the nascent molecule, resulting in new lipopeptides with various performances (11). Nonetheless, engineering natural product pathways has often yielded poor results, in part due to poor translation of *in silico* predictions to actual functional pathways and molecules (12, 13).

To tackle the difficulty of generating structural diversity of NRPs, we decided to integrate several distinct approaches in this work. Our goal was to build a single biosynthetic pathway capable of producing a diversity of new and structurally distinct molecules. Specifically, we combined precursor-directed biosynthesis, combinatorial genetics, and heterologous expression of biosynthetic genes to assemble new, unnatural NRPs in a programmable fashion. Precursor-directed biosynthesis enabled us to control the molecules that are made by providing the organism with precursors to be incorporated into the nascent molecule. By using combinatorial genetics, we expanded the diversity of the molecules that could be made through the use of alternative pathways, and by expressing these pathways heterologously, we limited background interference and enabled better control over production.

To increase the likelihood of success, we focused on iron-chelating nonribosomal peptides called siderophores (14). These molecules are key for cell survival under low soluble-iron availability. By linking the production of new molecules to survival, we sought to drive the organism to produce new molecules or otherwise perish. Specifically, we deconstructed the serratiochelin biosynthetic pathway and reconstructed a simple and reduced version incorporating only its biosynthetic genes. We also built an alternative pathway utilizing homologous genes from *E. coli* and *Vibrio cholerae* responsible for the biosynthesis of enterobactin (15, 16) and vibriobactin (17), respectively. With this alternative pathway, we explored whether these closely related genes could produce the target molecules, which would yield insights into the evolution of pathways and the exchangeability of homologous enzymes. This synthetic pathway was capable of generating not only natural molecules, such as serratiochelin and enterobactin, but also nonnatural molecules by incorporating exogenously supplied precursors. In summary, we demonstrated the use of heterologous biosynthetic pathways, coupled with lethal selective pressure and distinct precursors, to create an assortment of new and nonnatural NRPs.

## RESULTS

Serratiochelins are catechol siderophores produced by *Serratia plymuthica* V4 (18, 19). These siderophores utilize catechol moieties for iron coordination, obtaining them from the conversion of endogenous chorismate to dihydroxybenzoate (DHB) (19). The chorismate-to-DHB pathway appears to be extremely conserved among catechol siderophores (20). Additional enzymes can then use DHB to form a wide diversity of catechol-based molecules, such as enterobactin, fluvibactin, vibriobactin, photobactin, petrobactin, and vulnibactin (21).

We hypothesized that *E. coli*, which produces enterobactin (15, 16), could produce serratiochelins. This hypothesis was made based on several assumptions: (i) that the machinery responsible for the import and export of siderophores in this host would recognize serratiochelins and their catechol moieties; (ii) that *E. coli* could take up polyamines, such as diaminopropane (DAP) (Table 1), which are required for the production of serratiochelins and their nonnatural analogs; (iii) that the genes from the DHB pathway from *S. plymuthica* would be functional in *E. coli*, given that they are highly similar to the genes in the DHB pathway from *E. coli* (4, 19); and (iv) that expressing these pathways in a heterologous organism and under iron-limited conditions would allow us to supplement the medium with different precursors to generate new analogs.

**TABLE 1:** List of precursors, their reference numbers, final working concentrations, and incorporation in the new molecules

Polyamine precursor or dipeptide		Catalog no. (Sigma-Aldrich) or other supplier	Concn in medium	Incorporation in molecule	
No.	Name			Partial	Full
Polyamine precursors					
1	Diaminopropane	D23602	8 mM	Yes	Yes
2	Spermidine	S0266	8 mM	Yes	Yes
3	Spermine	S4264	1 mM	ND^a^	ND
4	Cadaverine	D22606	1 mM	Yes	ND
5	Putrescine	P5780	2.5 mM	Yes	Yes
6	Norspermidine	I1006	10 mM	Yes	Yes
7	*m*-Xylylenediamine	X1202	2.5 mM	Yes	ND
8	*N*,*N*′-Bis(2-aminoethyl)-1,3- propanediamine	333131	5 mM	ND	ND
9	*N*-Benzylethylenediamine	462292	2.5 mM	Yes	Yes
10	4-Aminobenzylamine	368466	2.5 mM	Yes	ND
11	4-(2-Aminoethyl)aniline	123056	0.5 mM	Yes	ND
12	4,4′-Oxydianiline	248398	0.05 mM	Yes	ND
13	4,4′-Diaminodiphenylmethane	32950	0.01 mM	ND	ND
14	1,5-Diaminonaphthalene	D21200	5 mM	ND	ND
15	2,2′-Thiobisacetamide	S365033	0.02 mM	ND	ND
16	Sulfaguanidine	S8751	2.5 µM	ND	ND
17	*p*-Aminobenzenesulfonamide	S9251	0.05 µM	ND	ND
18	Urea	U5378	5 mM	ND	ND
19	*N*-Phenylthiourea	P7629	5 mM	ND	ND
20	3,3′-Diamino-*N*- methyldipropylamine	188441	5 mM	ND	ND
21	1, 8-Diaminooctane	D22401	5 mM	Yes	ND
				
Dipeptides					
22	Dipeptide KR	Biomatik USA	0.01 mM	ND	ND
23	Dipeptide KK			ND	ND
24	Dipeptide KQ			ND	ND
25	Dipeptide QN			ND	ND

a ND, not detected.

**FIG 1:**
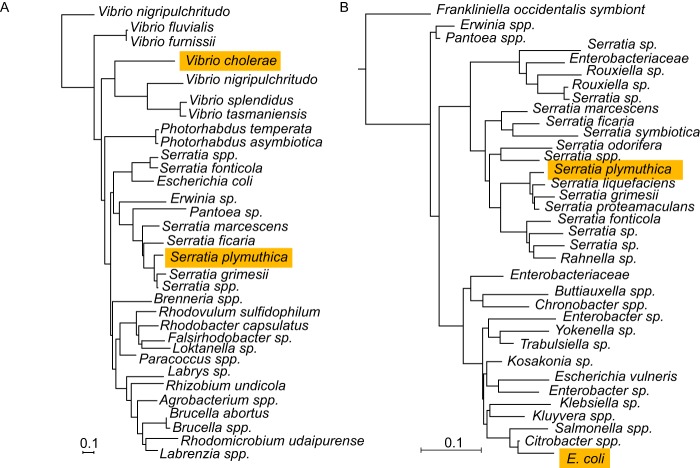
Phylogenetic trees displaying the relatedness of SchH and VibH (A) and SchE and EntE (B).

Initially, the *S. plymuthica* genes involved in the production of serratiochelins were cloned in a single operon into plasmid pDSW204 and driven by its isopropyl-β-d-thiogalactopyranoside (IPTG)-inducible promoter, which is a weaker version of promoter trc99A. This synthetic operon is a compressed version of the two-cluster serratiochelin biosynthetic pathway (Fig. 1A and B). It contained genes *schABCEG* from *S. plymuthica*. These genes are homologous to the enterobactin biosynthesis genes *entABCDE* from *E. coli* (16, 22, 23), with a protein identity of at least 57%, except for EntD, which shares an identity of 24% with SchG. The synthetic operon also contained *schF1F2F3*, which are homologous to *vibF* (51% protein identity), and *schH* (a *vibH* homolog; 32% protein identity). Genes *vibF* and *vibH* are involved in the biosynthesis of vibriobactin, a siderophore from *V. cholerae* (24–26). In addition to the large pathway, which we called SP_S, a *cos* site was also cloned for plasmid stability, and the resulting construct was named pSP_S.

The constructs were transformed into an *E. coli* Ent^−^ strain in which *entABCDEF* were deleted. *E. coli* Ent^−^ carrying pSP_S or the empty vector was grown under iron-deprived conditions at 30°C with agitation, in the presence or absence of DAP. This diamine is naturally produced by *S. plymuthica* (19). Growth was not observed under either of these conditions. This lack of growth could result from the inability of the *E. coli* machinery to express *S. plymuthica* gene clusters or the enzymes being inactive in *E. coli*. The enzymes responsible for assembling NRPs function in an assembly-line fashion (3), so if a single enzyme is not present or is nonfunctional, the ultimate target molecule will not be made.

**FIG 2:**
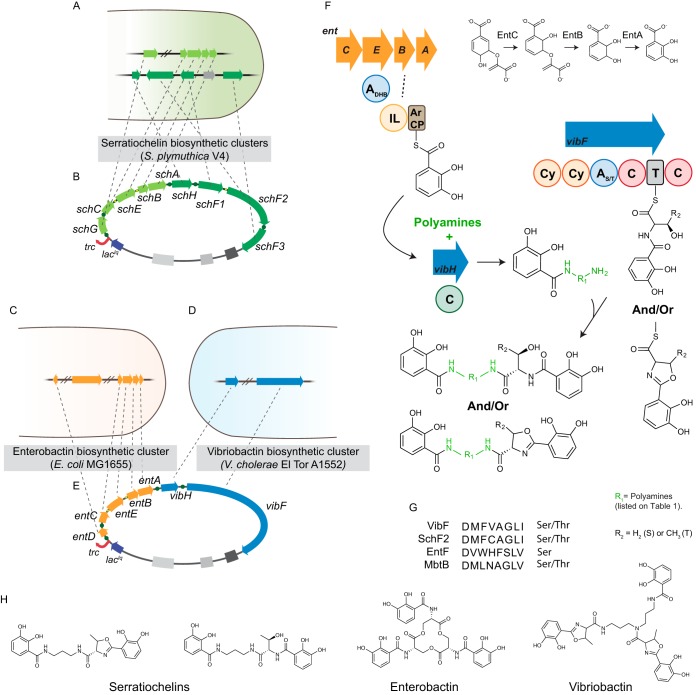
Compressed synthetic pathways for heterologous expression of natural and unnatural nonribosomal peptides. Heterologous expression of serratiochelins in *E. coli* Ent^−^ was initially attempted by cloning *S. plymuthica* biosynthetic genes (A) into a single operon, driven by an IPTG-inducible promoter (B). Upon failure to produce serratiochelins heterologously by expressing the *S. plymuthica* biosynthetic genes in *E. coli*, genes from *E. coli* MG1655 (C) and *V. cholerae* A1552 (D), which are homologous to those involved in the biosynthesis of serratiochelins, were cloned into a single operon (E). The biosynthetic processes for analogs are depicted in panel F. Chorismate is converted to DHB via a series of enzymatic reactions catalyzed by SchC/EntB, SchB/EntB, and SchA/EntA. DHB is then loaded onto the aryl carrier domain of EntB, for incorporation into the nascent molecule. l-Serine or l-threonine is activated by the adenylation domain of VibF and loaded onto the thiolation domain of the same enzyme. The amino acid can be further cyclized, as described elsewhere (19), thus increasing molecular diversity. VibH is responsible for condensing the intermediate molecules with the polyamines. Our results confirm *in silico* predictions for amino acid activation, where VibF, similarly to serratiochelin’s SchF2 and mycobactin’s MbtB, is predicted to activate l-threonine and l-serine as well, in contrast to enterobactin’s EntF, which activates only l-serine (G). The structures of serratiochelins, enterobactin, and vibriobactin are depicted in panel H.

To overcome this issue, we hypothesized that homologs of the *S. plymuthica* genes could produce a functional assembly line capable of synthesizing serratiochelins and new analogs. As noted above, the *schABCEG* and *schF1F2F3* operons are related to *E. coli* and *V. cholerae* genes that produce enterobactin and vibriobactin, respectively (Fig. 1). Also, it has been previously reported that holo-EntB, acylated with DHB by EntE, can serve as the substrate for the activity of VibH, similarly to VibB (26) (Fig. 2C and D). Thus, instead of cloning *S. plymuthica* V4 genes, we used their *E. coli* orthologs and *V. cholerae* ancestral homologs (19). Genes *entABCDE* and *vibFH* formed the pathway that we named EV_S and were assembled into the same empty pDSW204 backbone, along with a *cos* site, and introduced into *E. coli* Ent^−^ (Fig. 2C, D, and E) as a construct called pEV_S.

**FIG 3:**
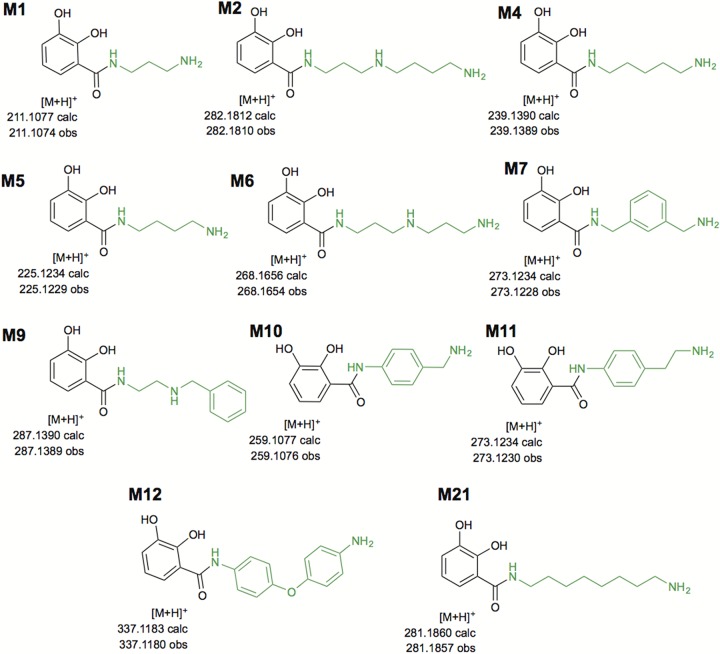
Proposed structures for the DHB-polyamine intermediates assembled by the compressed pathway. By adding various polyamines to the growth medium, VibH was found to be able to catalyze the reaction between foreign free polyamines (in green) and the tethered DHB. The [M + H]^+^ calculated and observed exact mass values for each molecule are also given. Each molecule is identified by the letter “M” and a number, corresponding to the polyamine added to the medium.

**FIG 4:**
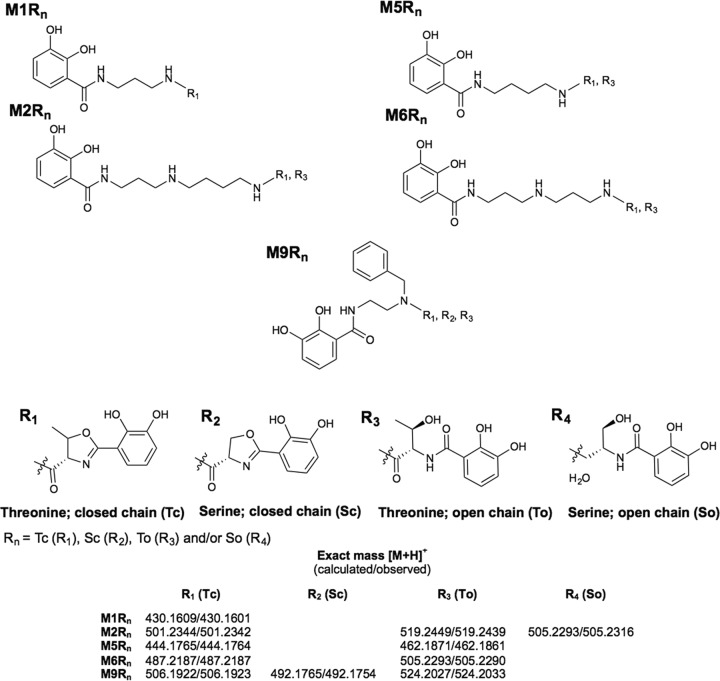
Proposed structures for the new serratiochelin analogs. The VibF acylation of the primary amine from the intermediates depicted in Fig. 3 can occur with 2-(2,3-dihydroxyphenyl)-5-methyloxazolinyl (R_1_ and R_3_) and/or a 2-(2,3-dihydroxyphenyl)-oxazolinyl (R_2_ and R_4_) as well. In some samples, the amino acid incorporated into the intermediate was found not to have gone through an additional cyclization, thus remaining in the open conformation as dihydroxybenzoyl-l-threonine and -serine (R_3_ and R_4_). Each molecule is identified by the letter “M” and a number, corresponding to the polyamine added to the medium, as well as the amino acid (S, serine; T, threonine) incorporated and its configuration (c, closed; o, open). R_n_ indicates the alternative radicals for the structures proposed and detected in the samples.

pEV_S enabled the growth of *E. coli* Ent^−^ under iron-limited conditions in the presence of DAP. Upon analysis of the Sep-Pak tC_18_-purified supernatant, we confirmed the production of the serratiochelin precursor (Fig. 3, M1, and see Fig. S1 at https://figshare.com/s/6238bd55b771b7853ff7) and the full-sized serratiochelin as well (Fig. 4, M1Tc, and see Fig. S2 at https://figshare.com/s/4abc2ac1669a6cb52a7b). However, we also observed growth of *E. coli* Ent^−^ carrying pEV_S in the absence of diaminopropane, suggesting that another siderophore could be assembled by the biosynthetic pathway independently of the polyamine supplemented. We investigated this unexpected observation by analyzing the tC_18_-purified supernatant, wherein we detected the production of enterobactin (see Fig. S3 at https://figshare.com/s/4e163eaf8f89add99333), as well as linear enterobactin (see Fig. S4 at https://figshare.com/s/c31b4a46cf2a0e73ba76) and its dimers and monomers (see Fig. S5 and S6 at https://figshare.com/s/68fbe7b23716cbc45e2f and https://figshare.com/s/3363ded7d1e0ffc7ef16, respectively). These data indicate that VibF can replace EntF to assemble enterobactin, thus enabling cells to grow even in the absence of the precursor diaminopropane. In addition to analyzing samples with no added amines for enterobactin production, we decided to check whether samples to which amines had been added—thus enabling serratiochelin analogs to be made—also produced enterobactin. Enterobactin was detected in all but two of these samples (see Fig. S7 at https://figshare.com/s/994abf66ce48070d920d). Linear enterobactin and its dimers and monomers were also found in most samples (see Fig. S8d at https://figshare.com/s/0c70fd870d8d66c9fa0d, S9d at https://figshare.com/s/16e58bba259c540abc08, and S10d at https://figshare.com/s/fdf582d9e0addf61666f). The fact that our engineered strains continued to produce enterobactin along with serratiochelin and its analogs suggests that the former molecule is less resource intensive to produce than the latter molecules: enterobactin does not require polyamines (or VibH) for assembly. *E. coli* Ent^−^, the control strain that did not contain the heterologous biosynthetic pathway, could not grow under the same conditions tested here.

Given this observation, we then asked whether this pathway could produce Thr-enterobactin analogs, which would confirm predictions by other groups (27, 28). This question is relevant because based on *in silico* analysis, the adenylation domain signature of VibF is predicted to activate l-threonine and l-serine, whereas that of EntF is predicted to activate l-serine exclusively (Fig. 2G). The signature in VibF is very similar to that of MbtB (Fig. 2G). MbtB is a part of the pathway that synthesizes the siderophore mycobactin in *Mycobacterium tuberculosis* (29). In other species of the genus *Mycobacterium*, this siderophore has been found to contain l-serine or l-threonine via MbtB activation (29–31). Regarding VibF, *in vivo* incorporation of l-serine was initially thought to be unlikely by some (24) but was subsequently observed *in vitro*, albeit at a low level (27, 28). Keating et al. (27) made this observation when l-serine was added as a supplement individually to the *in vitro* reaction and different amino acids were not available to be activated by the adenylation domain of VibF. In our experiments, only the Thr-enterobactin dimer (see Fig. S11 at https://figshare.com/s/3a9f8994f468e552ce09) and monomer (see Fig. S12 at https://figshare.com/s/b73a0a71a913c93f49a7) were detected and only in a reduced number of samples (see Fig. S13 and S14 at https://figshare.com/s/5f2475fd6487f7ff196d and https://figshare.com/s/85e9b17edbb34b86f05f, respectively). We cannot exclude the possibility that Thr-enterobactin was produced but at levels too low to be detected in some of the samples.

**FIG 5:**
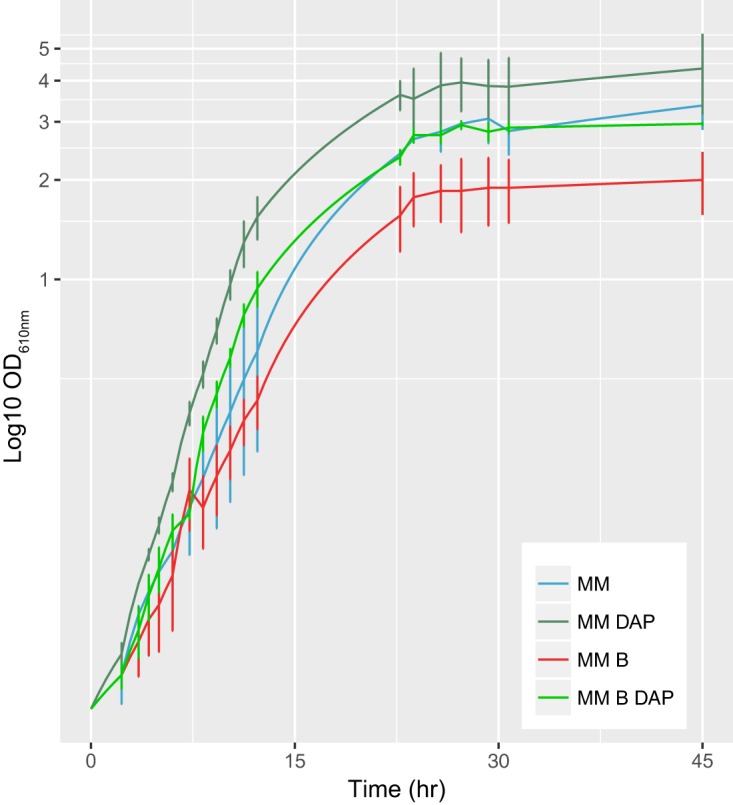
Growth over time of *E. coli* Ent^−^ pEV_S in minimal medium, in the presence of 0.1% bipyridyl and/or 8 mM DAP, measured as optical density at 610 nm over the course of time.

To the best of our knowledge, this is the first report of *in vivo* VibF l-serine activation to generate enterobactin, as well as l-threonine activation to produce a new Thr-enterobactin dimer. This alternative pathway for enterobactin assembly enabled bacterial survival under low-iron conditions and in the absence of externally supplemented polyamines. For this reason, survival was dependent on the biosynthetic pathway but independent of the assembly of serratiochelin analogs and might have led to the production of a lower variety of molecules due to a relief in the strong, lethal selective pressure that results from the unpredicted production of the iron chelator enterobactin. We could potentially engineer a VibF that would not incorporate l-serine but only l-threonine, or other amino acids, to avoid the production of enterobactin. Nonetheless, this would be expected to reduce diversity, as no l-serine molecules would be assembled. To assess whether we can enact selective pressure for the production of polyamine-dependent siderophores, we grew the engineered *E. coli* Ent^−^ carrying pEV_S in iron-depleted medium with bipyridyl (which chelates the little soluble iron available) and/or DAP (since the absence of DAP should make the cells rely on only enterobactin for iron uptake). We found that DAP enabled a higher growth rate in the presence of bipyridyl (µ = 0.357) compared to cells grown with bipyridyl alone (µ = 0.298, *P* = 0.02 [Fig. 5]). This suggests that the catechol molecules incorporating DAP are indeed siderophores. In fact, both serratiochelin (which incorporates DAP) and M5Tc/photobactin (which differs from serratiochelin only in its polyamine moiety, which in photobactin is putrescine) are known siderophores (19, 32).

Having designed and built a hybrid pathway that produces predicted molecules as a function of the precursor added (Fig. 2F), we wanted to determine the extent to which this approach could be adapted to create additional molecules. Polyamines with various numbers of carbons and amine groups, with and without other moieties, as well as four dipeptides, were independently added to the growth medium of *E. coli* Ent^−^ carrying pEV_S (Table 1). The concentration of polyamines used was determined as the highest concentration that would not inhibit the growth of the producer strain in the absence of iron.

The biosynthetic and programmable pathway was capable of generating several predicted intermediate NRPs in which a polyamine was condensed with DHB (Fig. 3). In fact, VibH condensed linear polyamines and also condensed aromatic ones, such as aminobenzylamine (M10, Fig. 2) and oxydianiline (M21, Fig. 3), forming intermediate molecules that are the base for full-sized analogs. The annotated tandem mass spectrometry (MS/MS) spectra for the proposed structures in Fig. 3 can be found summarized in Table 2 and fully described in the supplementary information at figshare (see Fig. S13 to S34 at https://figshare.com/s/43bb422cac98e3dfeebd). In addition, we observed that the capacity of the pathway to generate full-sized serratiochelin analogs seemed to be restricted mostly to linear polyamines containing up to 4 amine groups and 9 carbons (Table 1, polyamine 2; Fig. 4, M2R_n_). Nonetheless, VibF incorporated l-serine or l-threonine into these molecules and cyclized them or not, depending on the polyamine precursor supplemented. VibH was found to be very flexible in the substrate upon which it could act based on transferring activated DHB from EntB to a diversity of acceptor amines (Fig. 3; also Fig. S15 to S34 at https://figshare.com/s/43bb422cac98e3dfeebd).

**TABLE 2:** Exact mass and electrospray ionization-MS/MS fragmentation pattern for all molecules assembled^a^

Molecule	Exact mass (calc/obs)	Fragmentation (calc/obs)
1	211.1077/211.1073	137.0239/137.0231
		194.0817/194.0809
1Tc	430.1609/430.1601	137.0239/137.0230
		194.0817/194.0811
		277.1175/277.1188
		294.1443/294.1457
2	282.1812/282.1810	72.0813/72.0813
		129.1392/129.1388
		194.0817/194.0810
		265.1552/265.1542
2Tc	501.2344/501.2342	194.0817/194.0812
		265.1552/265.1540
		308.1610/308.1595
		365.2178/365.2173
2So	505.2293/505.2316	137.0239/137.0237
		209.0926/209.0924
		224.0559/224.0555
2To	519.2449/519.2439	137.0239/137.0232
		194.0817/194.0809
		210.0766/210.0758
		265.1552/265.1542
		282.1807/282.1809
		383.2283/383.2294
4	239.1390/239.1389	86.0970/86.0970
		103.1229/103.1235
		137.0239/137.0232
		222.1130/222.1132
5	225.1234/225.1229	72.0813/72.0815
		89.1068/89.1080
		137.0239/137.0234
		208.0974/208.0968
5Tc	444.1765/444.1764	137.0239/137.0229
		192.0661/192.0661
		208.0974/208.0964
		225.1228/225.1237
		308.1599/308.1617
5To	462.1871/462.1861	137.0239/137.0230
		208.0974/208.0964
		225.1228/225.1237
		238.0715/238.0717
		210.0766/210.0755
6	268.1656/268.1654	137.0239/137.0232
		194.0817/194.0810
		251.1396/251.1381
		443.1925/443.1907
6Tc	487.2187/487.2187	137.0239/137.0232
		194.0817/194.0810
		277.1188/277.1188
		351.2021/351.2016
		443.1925/443.1907
6To	505.2293/505.2290	137.0239/137.0232
		194.0817/194.0810
		210.0766/210.0758
		369.2127/369.2130
7	273.1234/273.1228	120.0813/120.0811
		137.0239/137.0232
		256.0974/256.0964
9	287.1390/287.1389	91.0548/91.0545
		180.0661/180.0651
9Tc	506.1922/506.1923	91.0548/91.0546
		180.0661/180.0652
		287.1385/287.1381
9Sc	492.1765/492.1754	91.0548/91.0548
		287.1385/287.1385
9To	524.2027/524.2033	91.0548/91.0548
		345.1439/345.1460
		389.1934/389.1956
10	259.1077/259.1076	106.0657/106.0656
		137.0239/137.0237
		154.0493/154.0497
11	273.1234/273.1230	120.0813/120.0813
		137.0239/137.0234
		256.0974/256.0963
12	337.1183/337.1180	108.0449/108.0446
		137.0239/137.0232
		201.1017/201.1024
21	281.1860/281.1857	128.1439/128.1435
		137.0239/137.0231
		145.1694/145.1699
		264.1600/264.1595
Ent	670.1515/670.1509	137.0239/137.02134
		206.0459/206.0452
		224.0553/224.0554
		447.1034/447.1029
Ent trimer	688.1621/688.1613	137.0239/137.0230
		224.0559/224.0555
		447.1040/447.1018
Ent dimer	465.1140/465.1133	137.02139/137.0231
		196.0610/196.0609
		224.0559/224.0557
Ent monomer	242.0659/242.0653	106.0493/106.0503
		137.0239/137.0234
Thr-Ent dimer	493.1453/493.1448	137.0239/137.02131

a calc/obs, calculated/observed; Ent, enterobactin.

We sought to determine whether the pathway could be used to assemble vibriobactin by adding norspermidine (Table 1, polyamine 6) to the medium. This molecule was not detected in the supernatant, but its intermediate with only the primary amines acylated was indeed detected (Fig. 4, M6Tc, and see Fig. S24 at https://figshare.com/s/f8395aa5e8853c1e70cb), as well as an additional analog, M6To (Fig. 4; see also Fig. S25 at https://figshare.com/s/fbfcb15ad60f1daf0ecd). This was not surprising, as others had already unsuccessfully tried to assemble vibriobactin *in vitro* (26).

One of the factors limiting the diversity of analogs generated appeared to be VibF, not VibH. Given that we detected several of the serratiochelin mutasynthon intermediates in the supernatant, VibF seems incapable of condensing its dihydroxyphenyl-5-methoxyxazoline (and l-serine-containing derivative) with the polyamine-containing intermediate. Several approaches could potentially enhance the performance of this synthetic pathway in terms of assembling full-sized molecules. For example, VibF could be subjected to directed evolution and other VibF/SchF1F2F3 homologs could be tested. It is also possible that the molecules were indeed assembled but could not be exported to the extracellular space that was assayed.

Interestingly, we observed what seemed to be a preferential orientation for condensing asymmetrical polyamines with the EntB-tethered DHB. For molecules M9 (Fig. 3; see also Fig. S25 at https://figshare.com/s/fbfcb15ad60f1daf0ecd) and M9Tc (Fig. 4; see also Fig. S26 at https://figshare.com/s/bb5ed59c88ba93b4aa59), a single fragmentation pattern, corresponding to that of a single orientation, was found. Nonetheless, we cannot exclude that the alternative conformation could exist at lower levels.

M5 corresponds to aminochelin (see Fig. S20 at https://figshare.com/s/49a77eddc280d6cc457f), a molecule that itself can act as a siderophore but can be incorporated into a large one, azotochelin, both produced by *Azotobacter vinelandii* (33, 34). We thus anticipate that the novel intermediate generated via this programmable pathway could possess metal-chelating abilities, similarly to its larger counterparts.

## DISCUSSION

Microorganisms display an extraordinary ability to synthesize molecules that have been used to target cancer cells, parasites, iron overload, and bacterial infections (35, 36). They have evolved sets of very large enzymes that interact to assemble acyl-CoA- or peptide-based molecules, PKs or NRPs, respectively. The genes encoding these enzymes are modular, and each module is responsible for the incorporation of one unit (3, 37). Productively tapping these pathways has nonetheless posed a great challenge to researchers. In addition to difficulties in cultivating some producer organisms, it can be challenging to find conditions that lead to novel molecule production or to engineer native or heterologous pathways for inducible or constitutive expression (38). Often, attempts to alter these pathways for the production of new molecules or to express them heterologously result in a complete shutdown of production (12, 39, 40).

Here, we report an approach for the effective production and structural diversification of molecules produced by a nonribosomal peptide synthesis pathway. We successfully used ancestral or ortholog biosynthetic genes, rather than original pathways, for the heterologous and programmable production of serratiochelins and new analogs. Given that most NRP synthetases (NRPSs) responsible for the assembly of a given molecule have evolved from preexisting NRPSs (20, 41, 42), we anticipate that this approach can be applied to other biosynthetic pathways for which ancestral or ortholog genes exist. We deconstructed the enterobactin and vibriobactin biosynthetic pathways, which are organized in multiple operons, and then reconstructed them into a single and hybrid pathway comprised only of the biosynthetic genes. Specifically, we cloned *entABCDE* and *vibFH* (24, 25) in a single operon whose expression was driven by an IPTG-inducible promoter into an enterobactin-deficient *E. coli* strain lacking *entABCDEF* (*E. coli* Ent^−^).

An assortment of structurally diverse NRPs was produced by supplementing the iron-deprived growth medium with different small-molecule precursors as the substrates for VibH. These molecules were analogs of serratiochelin and its intermediate. Through this approach, new molecules were generated by adding precursors to the medium. If there is a specific moiety that one desires to include in the nascent molecule, the corresponding precursor can be supplied to the medium for incorporation, as long as it contains at least one amine group and the biosynthetic enzymes can use the precursor as a substrate for activity. Additional structural diversity was generated due to the capacity of VibF to activate not only l-threonine but l-serine as well for incorporation into the nascent molecule. To the best of our knowledge, this is the first report of *in vivo* VibF activation of l-serine. Nonetheless, not all precursors could serve as a substrate to VibF or VibH, and even when they could, there seemed to be a slight preference for l-threonine over l-serine activation (Fig. 4). Nearly half of the precursors tested were incorporated into the nascent molecule, and over half of these led to additional new structures, as detected and recognized by liquid chromatography (LC)-MS/MS. This methodology has been routinely used for this particular type of application (11, 43–47). The MS/MS pattern of each new molecule is known because the biosynthetic process is well characterized for serratiochelins (19).

We found that our synthetic pathway successfully assembled the cyclic and linear versions of enterobactin, as well as its monomer and its dimer. It also assembled a new version of linear enterobactin, dimer, and monomers, containing not l-serine but l-threonine. The latter observation is in line with the theory of the evolution of gene collectives (20), which are suggested to be sets of genes that coevolved quickly to lead to new molecules with minimal effort. The fact that multiple siderophores are assembled, at least in part, by biosynthetic pathways that are phylogenetically related supports this theory. Here, we show that combining genes from independent pathways can lead to new molecules as well as known molecules that are assembled by different enzymes. Moreover, we also show how ancestral genes and genes can allow for heterologous expression of NRPs when the original genes do not. In addition, it was interesting to observe how the programmable pathway SP_S containing the serratiochelin biosynthetic genes was not capable of synthesizing iron chelators in *E. coli*, despite it being related to pathway pEV_S. The fact that their ancestors could make both serratiochelin and enterobactin (though not vibriobactin, but its intermediates), and additional derivatives, shows how using ancestor genes instead of the original ones can lead to the successful heterologous expression of molecules.

Algorithm-based analyses of these molecules suggest that they may have relevant properties with clinical applications (see Tables S1 to S3 at https://figshare.com/s/43bb422cac98e3dfeebd). For example, all molecules were predicted to be potentially drug-like (see Table S2 at the URL mentioned above) and well absorbed (violations, <2; see Table S3 at the URL mentioned above). These algorithms suggest that some of the molecules generated could potentially target G-protein-coupled receptors (GPCRs) and ion channel modulators, among others (see Table S2 at the URL mentioned above). Future cellular and *in vivo* work will be needed to characterize the therapeutic potential of these molecules and whether these predicted activities are real. Siderophores may also have nonclinical applications, such as bioremediation, given their capacity to bind divalent cations (e.g., Cd^2+^, Cu^2+^, Ni^2+^, Pb^2+^, and Zn^2+^), trivalent cations (Mn^3+^, Co^3+^, and Al^3+^) and actinides (e.g., Th^4+^, U^4+^, and Pu^4+^) (48). Thus, the new analogs of siderophores could be tested *in vitro* and assayed for their metal binding affinities and specificities.

In addition to biological testing of the newly assembled molecules, future work should focus on screening amide synthases for their substrate tolerance in order to try to further increase the diversity of molecules. Furthermore, in order to block the assembly of enterobactin, one could mutate the adenylation domain of VibF, specifically the 10-amino-acid signature of this domain that is responsible for amino acid activation (49–51). Given that these 10 amino acids are dispersed throughout the domain, we would expect that most of the mutants generated would result in complete loss of function instead of a threonine-only activator.

In summary, we envision that using ancestor genes for molecular assembly along with precursor supplementation could be used for the diversification of chemical entities of biological interest in tractable, heterologous organisms. This approach could be applied to other nonribosomal pathways for which heterologous expression has posed a problem and improved with *in silico* molecule design as well as high-throughput strategies for pathway optimization and precursor application.

## MATERIALS AND METHODS

### Strains and general growth media.

All *E. coli* strains and *S. plymuthica* were maintained on lysogeny broth (Miller; Lab Express) supplemented with 1.5% agar and appropriate antibiotics as required. *V. cholerae* was maintained on agar plates prepared with marine broth 2216 (BD Diagnostics). *Saccharomyces cerevisiae* was maintained on complete supplement mixture medium (CSM; Sunrise Science Products) or CSM-tryptophan dropout medium for selection and maintenance of the yeast artificial chromosome (YAC; pYES-1L) carrying the assembled pathways.

### Construction of an *E. coli* strain for heterologous expression of serratiochelins.

The genes responsible for serratiochelin production are distributed between two gene clusters in *S. plymuthica*. One of these clusters contains two genes homologous to those involved in the production of vibriobactin (in *V. cholerae*). The other contains 6 genes that are homologs to those involved in the production of enterobactin (19). These 6 homologs were removed from the chromosome of *E. coli*, given that our goal was to express serratiochelin and its analogs in this organism without their interference. The new *E. coli* strain was called *E. coli* Ent^−^. This was done so that the extrachromosomal synthetic pathway was responsible for molecular biosynthesis. Gene deletion was achieved using the Lambda Red recombination system (52); *entD*, *entCEBA*, and *entF* were replaced with chloramphenicol, kanamycin, and gentamicin resistance, respectively. The removal of the enterobactin biosynthetic genes disables this organism’s capacity to assemble this siderophore and grow under iron-limited conditions (16, 53, 54).

### Construction of compressed synthetic pathways for the assembly of serratiochelin analogs.

The serratiochelin biosynthetic genes *schCEBA* (GenBank accession numbers AHY08568.1, Sch_19080, AHY08567.1, and AHY08566.1), *schF1F2F3* (GenBank accession numbers AHY05890.1, AHY05889.1, and AHY05892.1), *schG* (GenBank accession number AHY08579.1), and *schH* (GenBank accession number AHY05888.1) were cloned from *S. plymuthica* V4 (identifier [ID] CP007439.1), which produces serratiochelins. The enterobactin genes *entCEBA* (IDs 945511, 947426, 946178, and 945284) and *entD* (ID 945194) are from *E. coli* MG1655 (ID NC_000913.3). *vibF* (ID 2614958) and *vibH* (ID 2615318) are from *V. cholerae* El Tor A1552 (ID N16961) (Table 3). Genes *schCEBA*, *entCEBA*, and *schF1F2* were cloned as open reading frames and operons. The *E. coli* ribosome binding site (RBS) GAGGAGA was placed upstream of genes *schC*, *entC*, *schF1*, *schH*, *vibH*, *schF3*, *vibF*, *schG*, and *entD*.

**TABLE 3:** List of bacterial strains and their genotype and/or phenotype and source, as well as plasmids used and built, and their characteristics and source

Strain/plasmid	Genotype/phenotype/description	Source or reference
Strains		
*E. coli* Top10	Large plasmid cloning strain, F^−^ *mcrA* Δ(*mrr*-*hsdRMS*-*mcrBC*) Φ80*lacZ*ΔM15 Δ*lacX74 recA1 araD139* Δ(*ara leu*)*7697* *galU galK rpsL* (Str^r^) *endA1 nupG*	GeneArt Life Technologies, Inc.
*E. coli* DH5α	Cloning strain, F^−^ Φ80*lacZ*ΔM15 Δ(*lacZYA-argF*)*U169* *recA1 endA1 hsdR17* (r_K_^−^ m_K_^+^) *phoA supE44* λ^−^ *thi*-*1 gyrA96 relA1*	Laboratory collection
*E. coli* K-12 MG1655	Wild type and enterobactin producer, F^−^ λ^−^ *ilvG rfb-50 rph-1*	Laboratory collection
*E. coli* Ent^−^	MG1655 Δ*entD*::Cam^r^ Δ*entCEBA*::Kan^r^ Δ*entF*::Gent^r^	This study
*Serratia plymuthica* V4	Serratiochelin producer (ZK4911)	19, 71
*Vibrio cholerae* O1 El Tor A1552	Wild type, O1 El Tor Inaba, vibriobactin producer	Laboratory collection
*Saccharomyces cerevisiae* MaV203	*MAT*α *leu2-3,112 trp1-901 his3*Δ*200 ade2-101 gal4*Δ *gal80*Δ *SPAL10*::*URA3 GAL1*::*lacZ* *HIS3*_UAS GAL1_::*HIS3*@*LYS2 can1*^R^ *cyh2*^R^	GeneArt Life Technologies, Inc.
		
Plasmids		
pYES-1L	Yeast artificial chromosome, *S. cerevisiae*-*E. coli* shuttle vector, Trp^−^ Spec^r^	GeneArt Life Technologies, Inc.
pDSW204	*E. coli* replicative expression vector with a medium-strength promoter, IPTG inducible	72
pWEB-TNC	*E. coli* cosmid, donor of the *cos* site	Epicentre
pEV_S	pDSW204 carrying pathway EV_S, with genes *entABCDE* and *vibHF* and a *cos* site	This study (Addgene plasmid #100266)
pSP_S	pDSW204 carrying pathway SP_S, with genes *schABCEF1F2F3GH* and a *cos* site	This study (Addgene plasmid #100270)

In addition to building the synthetic *sch*-based compressed pathway, containing the *S. plymuthica* genes *schABCEF1F2F3GH*, we also assembled a pathway from the *E. coli* enterobactin and *V. cholerae* vibriobactin genes. Given the homology between these two clusters of genes and those of the serratiochelin biosynthetic pathway, it is of interest to determine whether, when together, these genes can assemble serratiochelin. The degree of similarity between homologous proteins was assessed utilizing the BLAST blastp suite from the National Center for Biotechnology Information (55–57) and has also been addressed elsewhere (19).

The genes or open reading frames were PCR amplified with overhangs homologous to the genes to be located up- and downstream, in the compressed pathway. SpeI sites were added at both ends of each pathway, to allow the pathways to be released by restriction enzyme digestion from the cloning vector, a yeast artificial chromosome. The amplicons were transformed, assembled into full pathways in *S. cerevisiae* using the GeneArt high-order genetic assembly kit (Life Technologies, Inc.), and checked for proper assembly by PCR. The compressed pathways (Fig. 2B and E) were released from pYES-1L by restriction digestion with SpeI. Each of the inserts was cloned into pDSW204, to which a *cos* site (for large construct stability) and a SpeI site had been added. After being checked for proper assembly, the constructs were moved to *E. coli* Ent^−^ for production of serratiochelin and its analogs. The constructs are available on Addgene for distribution.

### Selection of exogenously supplied precursors.

To test the substrate limits for VibH, we offered several amine-containing small molecules as DHB acceptors. We selected precursors with the goal of generating a wide diversity of molecules with a range of chemical properties. All polyamine precursors were purchased from Sigma-Aldrich; dipeptides were synthesized by Biomatik. All precursor polyamines were either primary or secondary amines. The product references, names, and concentrations used are listed in Table 1.

Both the compressed and the hybrid pathways, introduced into *E. coli* Ent^−^, were tested for production of serratiochelin and vibriobactin. To this end, diaminopropane (incorporated in Fig. 3, M1, and Fig. 4, M1Tc) and norspermidine (incorporated in Fig. 3, M6, and Fig. 4, M6Tc/To) were added to the medium, respectively. To test whether other analogs could be generated, we selected other polyamines to be added to the growth medium. We chose a variety of molecules with up to 12 carbons and 4 amine groups [cadaverine, incorporated in Fig. 3, M4; putrescine, incorporated in Fig. 3, M5, and Fig. 4, M5Tc/To; spermidine, incorporated in Fig. 3, M2, and Fig. 4, M2Tc; 1,8-diaminooctane (incorporated in Fig. 3, M21), 3,3′-diamino-*N*-methyldipropylamine, and *N*,*N′*-bis(2-aminoethyl)-1,3-propanediamine], because they are similar to diaminopropane and norspermidine. 2,2′-Thiobisacetamide has two amides, which could potentially contribute for metal chelation, in addition to being a substrate for condensation by VibH. Furthermore, we selected two molecules, sulfaguanidine and *p*-aminobenzenesulfonamide, with structural similarity to synthetic sulfonamide antibiotics. Siderophores are taken up by cells using specialized transporters (14), and antibiotics that have evolved to structurally resemble siderophores (58) can take advantage of this mechanism to kill bacterial cells. Urea and *N*-phenylthiourea were also tested.

Putrescine, spermidine, cadaverine, and aminopropylcadaverine are polyamines naturally occurring in *E. coli* (59–63); although their molecular functions are not fully understood (64), there is evidence for a role in mRNA translation (63). We supplied these compounds exogenously in hope that they would be incorporated into unnatural nonribosomal peptides (NRPs). We expected that endogenous levels would be too low to contribute to molecule assembly (65).

Additional precursors were chosen with the potential to enhance fluorescence (66, 67) of the products. *m*-Xylylenediamine, *N*-benzylethylenediamine, 4-aminobenzylamine (incorporated in Fig. 3, M10), 4-(2-aminoethyl)aniline (incorporated in Fig. 3, M11), 4,4′-oxydianiline (incorporated in Fig. 3, M12), 4,4′-diaminodiphenylmethane, and 1,5-diaminonaphthalene contain one or two benzene rings in addition to the required amine groups. Fluorescent molecules can be tracked as they move into and out of the cell and could be used, for example, as an Fe^2+^ sensor in the medium, since bacteria will secrete the iron chelator only under low-soluble-iron conditions (68).

The antimicrobial activity of short peptides, such as KR-12, has been established against some bacteria (69). Efficient antimicrobial activity is correlated with inclusion of positively charged amino acids. To explore the potential for synthesizing antimicrobial peptides using one of our pathways, four dipeptides—lysine-lysine (KK), lysine-arginine (KR), lysine-glutamine (KQ), and glutamine-asparagine (QN)—were selected for testing as precursors.

### Production and purification of hybrid unnatural NRPs.

Minimal medium optimized for the production of serratiochelins (19) was used for molecule production by the synthetic pathways introduced into *E. coli* Ent^−^. It was composed of Na_2_HPO_4_ (5.96 g/liter), K_2_HPO_4_ (3.0 g/liter), NH_4_Cl (1.0 g/liter), NaCl (0.5 g/liter), MgSO_4_ (0.058 g/liter), C_6_H_12_O_6_ (5.0 g/liter), and IPTG (1 mM), pH 7.0. Precursors were added to final concentrations of 0.05 µM to 10 mM (Table 1). Siderophore production and related machinery were further induced by adding the iron chelator 2,2′-bipyridyl (Sigma-Aldrich catalog no. D216305) to a final concentration of 0.1 mM to the growth medium. In wild-type *E. coli*, bipyridyl activates the PentC promoter, which drives the expression of *entCEBA* (70). Cultures were grown to an optical density (600 nm) of ~2.8 (glucose depletion), for up to 7 days at 30°C with 250 rpm shaking.

After growth, cells were spun down and the supernatant was filter sterilized. Cell-free supernatant was loaded onto Sep-Pak tC_18_ (5 g) reversed-phase columns (Waters). The columns were washed with water, and the adherent molecules were eluted with 100% acetonitrile.

Liquid chromatography and tandem mass spectrometry (LC-MS/MS) sample analysis were performed at the Small Molecule Mass Spectrometry core facilities at Harvard University. Two-hundred-fifty-microliter aliquots of each sample were injected into a high-resolution, accurate mass Q Exactive Plus Orbitrap, with positive ionization and mass scan ranging from 66.7 to 1,000.0 *m/z* (resolution 70,000 FWHM [full width at half maximum]), and operated over the course of 30 min at a flow rate of 0.8 ml/min, with a gradient of 10% acetonitrile (ACN) in H_2_O to 100% ACN. Molecules displaying masses matching the expected ones were fragmented (35,000 FWHM), and the respective fragmentation patterns were compared against those of the predicted structures.

Structure predictions were performed based on previous knowledge of the NRP-based assembly of serratiochelins (19). We anticipated that, instead of condensing diaminopropane, VibH (much like SchH) would potentially incorporate the polyamines provided and VibF (like SchF1F2F3) would finalize the assembly of serratiochelin analogs.

Predicted structures and exact mass values, for the natural and unnatural molecules potentially assembled, were drawn and calculated using ChemDraw Professional 10 (PerkinElmer).

### Bacterial growth rate determination.

We determined how the producer strain *E. coli* Ent^−^ pEV_S grew in the presence of bipyridyl with and without DAP. For this, three independent experiments were performed, where 50 ml of minimal medium containing 0.1 mM bipyridyl only or 0.1 mM bipyridyl plus 8 mM DAP were inoculated with overnight cultures under the same conditions to an optical density (610 nm) of 0.05. Growth was followed until the cultures reached stationary phase. The growth rate for each growth curve was calculated using the R package *growthrates*.

### SchE and SchH phylogenetic trees.

Despite having already been discussed in an earlier work (19), we queried the NCBI database for 500 homologs of SchE and SchH, in order to show the shared origin of these enzymes and EntE and VibH, respectively. The Newick trees (neighbor joining, 0.85 maximum sequence difference, Grishin distance) were generated and downloaded from NCBI.

### Data availability.

The supplemental materials and methods, as well as supplemental data, can be accessed at figshare (https://figshare.com/s/43bb422cac98e3dfeebd).
